# Evidence-based modalities in the management of Psoriasis and Psoriatic arthritis

**DOI:** 10.6026/97320630019679

**Published:** 2023-06-30

**Authors:** Chiappelli Francesco

**Affiliations:** 1 Professor Emeritus, Center for the Health Sciences, UCLA, Los Angeles, CA; Dental Group of Sherman Oaks, CA 91403, USA.

**Keywords:** Evidence-based healthcare, Systematic review, Metascience, PICO(TS) question, Bibliome, Research synthesis, Translational evidence-based intervention, Psoriasis, Psoriatic arthritis (PsA), Methotrexate (MTX)

## Abstract

Psoriasis is a waxing and waning skin disorder, often associated with a plethora of co-morbidities, including psoriatic arthritis (PsA), a severe form of chronic inflammatory arthritis. All forms of psoriasis and PsA are immune-mediated diseases where the patient's immune system is overactive in the production of certain factors that stimulate and activate the function of certain immune cells. Recent evidence has uncovered an important role for cell-mediated immunity in the aetiology and course of psoriasis and PsA, with a critical role played by the pro-inflammatory IL-23/TH17 axis. Taken together, these new lines of evidence suggest new and improved therapeutic interventions for patients with psoriasis and PsA. The hypothesis-driven process of inquiry of the best available evidence and its implication, application and evaluation in the context of clinical practice pertains to the meta-science of evidence-based health care (EBHC). EBHC consists in the initial step of research synthesis and generation of the systematic review of the best available evidence, estimated both qualitatively and quantitatively (i.e., meta-analysis). Evidence-based decision-making, a process driven and controlled by the expertise of the clinician and by the clinical needs and personal wants of the patient, is the principal, most timely and critical aspect of evidence-based practice. Recent and systematic reviews for the treatment of psoriasis and PsA consistently updated for emerging new and revised data (i.e., living systematic reviews) confirm the efficacy and the effectiveness of methotrexate (MTX) in containing and controlling psoriasis. The outcomes of MTX intervention for PsA remain mixed and inconclusive.

## Background:

In the context of modern contemporary translational healthcare, that is to say the integration of scientific discoveries made in the laboratory in the clinical setting, the transformation of research findings into new treatments and medical interventions with the goal of improving the health of the population, it is timely and critical to make the clear distinction between evidence-based clinical modalities and approaches that are based on the evidence [1, 2, 3]. The term evidence-based health care (EBHC) refers specifically to clinical interventions that integrate the efficacy, effectiveness and efficiency of the best available evidence, produced by a systematic process of research synthesis, quality assessment and statistical evaluation. EBHC is initiated by assessing the clinical needs of the individual patient, and driven by the search of the optimal treatment intervention for the patients, or a sub-population of similar patients. In that sense, EBHC is patient-centered. Because EHBC is designed to obtain the most effective treatment intervention, it is, by design, effectiveness-focused. And, by the very nature of its process of inquiry, EBHC is evidence-based. By contrast, clinical approaches that are based on the evidence are typically limited and biased because they evolve from a limited view of the pertinent scientific literature. They do not, by definition, pertain to EHBC.

Here, we discuss evidence-based modalities of psoriasis and psoriatic arthritis (PsA). Psoriasis is a waxing and waning disorder of the integumentary system where the life cycle of skin cells is accelerated and deregulated, leading to a buildup of dead cells on the surface of the epidermis. This process leads to severe rashes with itchy, inflamed scaly patches and erosions that may crack and bleed, typically, on the knees, elbows, trunk, back and scalp. Psoriasis typically flares for a few weeks or months followed by periods of remission. It is more common in adults and the aging population than in children and young adults, and affects equally men and women. Psoriasis typically manifests as:

[1] Plaque psoriasis appears generally in adults and the elderly as raised, red patches of skin covered by silvery-white scales primarily on the scalp, trunk, and limbs, especially the elbows and knees.

[2] Guttate psoriasis appears primarily in children and young adults as small, red dots, typically on the torso or limbs.

[3] Pustular psoriasis appears across all age groups as pustules surrounded by inflamed red skin, usually affects the hands and feet.

[4] Inverse psoriasis appears mostly in pre- and peri-menopausal women as smooth, red patches in folds of skin, such as beneath the breasts or in the groin or armpits.

[5] Erythrodermic psoriasis appears rarely, but is a severe form of psoriasis characterized by red, scaly skin over most of the body, and generally as a sequela of another form of uncontrolled psoriasis.

Psoriasis is an erythemato-squamous disease associated with much comorbidity, including psoriatic arthritis (PsA) a severe form of chronic inflammatory arthritis that manifests initially as morning joint stiffness, and quickly progresses to stiffness, swelling and acute pain. PsA can affect any and all include joints, and characteristically flares rapidly and may unexpectedly subside. PsA is considered clinically as the most destructive form of arthritis. Other clinically significant sequelae of psoriasis include cardiovascular disease (e.g., heart attacks, strokes), kidney disease, liver disease, Type II diabetes, metabolic syndrome, obesity, osteoporosis (i.e., decrease in bone mass and mineralization), osteitis (i.e., intrinsic inflammation of the bones), onychodystrophy (i.e., abnormal changes in the shape, color, texture, and growth of the fingernails or toenails: nails may appear thick, ridged and pitted, with horizontal lines, deformity, discoloration, onycholysis [i.e., lifting of the nail plate from the nail bed], brittle, crumbling or splintering), dactylitis (i.e., severe swelling of fingers and toes), uveitis (i.e., inner inflammation of the eye), as well as mental health problems (e.g., anxiety depression).

## Psoriasis and Psoriatic Arthritis:

All forms of Psoriasis, and PsA are immune-mediated diseases where the patient's immune system is overactive in the production of certain factors that stimulate the multiplication of skin cells up to 10 times faster than normal, leading, as noted, to inflamed, scaly, itchy and oozing patches of cells erupting on the skin. The etiology psoriasis can be attributed to genetic (i.e., patients may have a family history of the condition), environmental (i.e., certain toxic environmental factors may exacerbate skin lesions or abrasions, which can then evolve into psoriatic manifestations), metabolic (i.e., conditions like the metabolic syndrome, fatty liver, or diabetes may favor and accelerate the onset of psoriasis), infectious diseases (i.e., certain bacterial [e.g., streptococcal] and viral infections [e.g., HIV] may precipitate the development of, or aggravate psoriatic lesions), addictions (e.g., smoking, drinking), pharmaceutical (e.g., certain medications for heart, parasitic or mental diseases can induce psoriatic lesions in some patients), and psychosomatic causes (e.g., psycho-emotional stress, psychological distress) [4, 5, 6, 7, 8].

## Immunopathology in Psoriasis:

The immunopathology of psoriasis manifests as a moderate to severe T lymphocyte-mediated immune pathogenesis of the skin. It is characterized by activation of innate immune cells (e.g., natural killer [NK] cells) and pathogenic T cells result in inflammatory response localized in skin tissue, and hyper-proliferation of keratinocytes. B cells, thought until recently to have a minor role in the pathogenesis of psoriasis, play an important part in the course of psoriasis since regulatory B cells (Bregs) that produce interleukin (IL)-10 seem to ameliorate psoriatic outbreaks [9]. T helper (H)1 and TH17 lymphocytes work in concerts with innate immune cells and cytokines in the pathogenesis of psoriasis through the release of inflammatory cytokines that promote further recruitment of immune cells, keratinocyte proliferation and sustained inflammation [10]. In brief, psoriasis is a Thelper cell-mediated inflammatory skin disease, where TH1, TH17 and TH22 sub-populations appear to play a prominent n important role. Circulating blood levels and titer in skin lesions of TH1 cytokines (e.g., IFNγ, IL-2, TNFα) and TH17 cytokines (e.g., IL17A, IL17F, IL22, IL26) are significantly increased in active psoriasis, as well as eruptive lesion IL23, IL20 and IL15 [11]. It is important to note that TH17 plays an important immune regulatory role, when activated by transforming growth factor-β (TGFβ) and IL6, including promoting mucosal defense, tissue integrity and curtailing immunopathogenic responses. By contrast, IL23-activated TH17 cells promote chronic tissue inflammation during infection, granuloma formation and autoimmunity [12]. In that light, the IL-23/TH17 pathway is known to plays a dominant role in psoriasis immunopathogenesis, and it may not be surprising that clinical data increasingly show promise for IL17- and IL23-specific antibody species for treating immune-mediated inflammatory diseases, including psoriasis [10, 11, 12, 13]. The critical role of the IL-23/TH17 axis in the pathogenesis of psoriasis-like lesions was confirmed experimentally in a murine model of psoriasis [14]. To be clear, the murine psoriasiform inflammation (PI) models have demonstrated the central role of the IL23/IL17 axis in psoriatic histopathology. Data show that these these cytokines mediate and regulate the long-lived, skin-resident innate lymphocyte and lymphoid cells, including T cells (CD3+TcR[αβ]CD4+/CD8+), and NK cells that accumulate in psoriatic lesions. The latter subpopulation is endowed with both overlapping and unique functions compared to circulating antigen-restricted αβ T lymphocytes. Murine PI is key to yielding a more complete understanding of the cross-modulatory signals that that bridge and regulate these distinct innate and adaptive immune cell types in the immunopathology of psoriasis for the development of new and improved targeted immunotherapies that mitigate or prevent disease progression [15].

As clear a role of inflammatory cytokines appears to be in psoriasis, it must not be overlooked that this skin pathology is a complex disease triggered by genetic, psychosomatic and environmental stimuli that work in concerns to mediate the observed immune deregulation, suggesting the need for a holistic approach in the development of psoriasis therapies. Certain genes have been linked to psoriasis, including the cluster of psoriasis susceptibility genes, some of which control and regulate keratinocyte biology and epidermal barrier function [16, 17].

Case in point, the human leukocyte antigen (HLA)-Cw6 allele confers the greatest psoriasis risk, albeit with a lower prevalence among Asians, in which HLA-Cw1 allele may more prevalent. In general, the HLA-Cw6 allele is predictive of type I early-onset psoriasis, guttate psoriasis, Koebner phenomenon (KP), susceptibility to the IL23/IL17 axis dysfunction and thus better response to immunotherapies targeting IL17, IL23 and related cytokines, and better response to conventional methotrexate (MTX) therapy. But, the HLA-Cw1 allele is predictive of erythrodermic psoriasis, pustular psoriasis, and axial PsA. The HLA-Cw1 allele is also associated with a lesser response to conventional therapies, in part because the etiology of psoriasis in individuals with the HLA-Cw1 allele appears to be, putatively, linked to concomitant viral agents, including cytomegalovirus [16, 17].

Furthermore, recent data suggest that certain HLA alleles may impede the effectiveness of psoriasis treatment. Case in point, the allele HLA-A Bw4-80I appears to be associated with difficult-to-treat psoriasis and reduce the probability of a beneficial treatment outcome. It is has been postulated that, mechanistically, this effect may be obtained because individuals with this particular allele may produce more TNFα and neutralizing NK activity through a predominance of activating HLA class I ligands and killer-cell immunoglobulin-like receptors, compared to patients with different alleles [18].

Skin abrasions can lead to KP-mediated generation of injured keratinocytes, which in turn promote abnormal calcium metabolism and possible epigenetic changes that contribute to the development of psoriatic lesions. Altered keratinocyte proliferation and differentiation is likely to be mediated by deregulated inflammatory cytokines (e.g., IL-23/TH17 axis), as well as invading immune cell populations. But, it is also a fact that keratinocytes, once activated, can contribute significantly to the secretion of anti-microbial peptides, of cytokines, as well as of chemokines. These factors can then act in concert with the products of immune cells in the psoriatic lesions to help initiate and reinforce inflammatory feedback loops. Psoriatic keratinocytes also show intrinsic differences (e.g., as noted, abnormal calcium metabolism, epigenetic alterations that promote psoriasis) from normal keratinocytes even after removal from the in vivo inflammatory environment. These observations are confirmed by murine PI models, which confirm the importance of keratinocytes in the etiology of psoriasis [19].

## Immunopathology in Psoriatic Arthritis:

Epidemiological data suggest that 1-3% of the population in Western societies are afflicted with psoriasis, and that close to one third of these patients develop as well a clinically significant form of psoriasis-related inflammatory arthritis, or spondyloarthropathy (i.e., most often HLA-B27-associated autoimmune arthritis localized at the spine and the joints of the arms and legs, and potentially involving the intestines and eyes as well), referred to as psoriatic arthritis (PsA) [20]. In fact, skin and nail lesions precede joint symptoms in more than 75-80% of patients with PsA [21].

Onycho-pathology (i.e., nail abnormalities) observed is psoriasis is a good predictor of the onset and course of PsA. Enthesitis (i.e, inflammation at the site of integration of tendons and bones) is a primary sign of PsA, and, there is a close anatomical relationship, for example, between the finger nail and the enthesis of the distal interphalangeal extensor tendon [i.e., nail root], one of the main entheseal compartments affected in psoriatic arthritis. In brief, the etiology of PsA is an auto-inflammatory process of enthesitis, associated with adjacent osteitis (i.e., inflammation of the bone matrix) and synovitis (i.e., inflammation of the synovium, or stratum synoviale, the connective tissue that lines the inside of the joint capsule), that is particularly damaging to affected joints and insidious, because fast propagating, to otherwise normal joints [21,22,23, 24]. As noted, the HLA-Cw6 allele is a strong predictor of psoriasis risk [16]. But, interestingly, genetic data show that HLA-Cw6 does not predict PsA-associated spondyloarthropathy and onycho-pathology, and confirm that PsA results from auto-inflammatory enthesitis, rather than autoimmunity [23].

The immunopathology of PsA seems to have similar culprits as psoriasis, namely T cells and inflammatory cytokines predominantly of the IL-23/TH17 axis [20, 24, 25]. However, interventions (e.g., nonsteroidal anti-inflammatory drugs, TNFα-inhibitors, including adalimumab, etanercept or infliximab) designed to block these cytokine pathways effectively in psoriasis, are less successful in treating PsA manifestations, perhaps because the precise mechanisms underlying the pathogenesis of PsA are multi-factorial, involving genetics, environmental factors, and immune-mediated inflammation, whose interactions and inter-relationship remain poorly understood [24,25,26,27,28]. Newer generation monoclonal antibody immunotherapeutics (e.g., the IL23 inhibitor guselkumab) have recently emerged as more effective treatment interventions for PsA, and less risk of side-effects including impaired mucosal immune surveillance in the oro-pharyngeal cavity (i.e., candidiasis) and inflammatory bowel disease [29]. Case in point, subcutaneous administration of the human monoclonal antibody guselkumab selectively blocks IL23 through binding to its IL23αp19 specific subunit, which binds to IL12p40, shared with IL12, to form IL23 [30], considered to be an effective blocker of wide-ranging auto-inflammatory flare-ups, as in inflammatory bowel disease [31], is now the first selective IL-23 inhibitor FDA approved to treat adults with active PsA [32].

## Evidence-Based Healthcare (EBHC):

EBHC is a product of metascience, the hypothesis-driven process of systematic inquiry of the best available evidence, and its optimal application in practice [33,34]. It integrates the best available evidence obtained by the systematic synthesis of evidence-based research into evidence-based practice. It is regarded as the optimal process to inform researchers and clinicians of the best available evidence in and evidence-based medicine [35, 36], evidence-based dentistry [37, 38], evidence-based nursing [39,40, evidence-based psychotherapy [41], and even evidence-based healthcare coaching [42, 43].

## Evidence-based research:

The best available evidence is derived systematically from the stringent, hypothesis-driven process of research synthesis, and is reported as systematic reviews [33, 42]. Across any field of inquiry, research evidence usually results from excellent science, but it may also emerge from scientific reports that suffer from serious methodologically flaws and biases. Research synthesis systematically sifts out the scientific evidence that is corrupt and that should not be utilized in evidence-based practice. It pulls together the consistent and coherent high-quality science; and it generates a qualitative and quantitative (i.e., meta-analysis) consensus of the best evidence for translation and integration into new and improved evidence-based practice [1,3, 33, 44 45]. In brief, EBHC in general, and evidence-based research in particular is effectiveness-focused because risk(cost)/benefit concerns must always remain primary. It is client/patient-centered because it is initiated by a research question that is centered on the patient's needs and wants.

Research synthesis in EBHC always commences with the PICO(TS) question: acronym for patient population (P), intervention (I), comparison (C), clinical outcome (O), timeline (T) and clinical setting (S). The very nature of the PICO(TS) question impart the most important client/patient-centered nature of research synthesis [for review, 1,3,33,46]. The PICO(TS) question is refined through the analytical framework [46] and validated by at least two independent assessors. Key words and phrases are generated that serve to gather all of the available research evidence and to refine the bibliome - the peer-reviewed publications directly pertinent to PICO(TS). Because research in any given field is constantly evolving, any one bibliome is in constant flux, and research synthesis reports - systematic reviews - require regular updating - hence, we speak of the best available evidence in living systematic reviews [45,46,47]. Each individual report in the bibliome is assessed and evaluated for the level of the evidence (i.e., type of study design, sampling bias), and the quality of the evidence (i.e., adherence to the principles of study design, methodology and statistical inference). A qualitative and quantitative (i.e., meta-analytical) consensus of the best available evidence obtained [1,3, 44,48]. The seamless transition from the outcomes of evidence-based research and research synthesis in translational healthcare models integrates evidence-based research consensus into evidence-based clinical practice [1, 3, 38, 44,49].

## Conclusion:

In brief, evidence-based modalities in Psoriasis and PsA can be summarized as the seamless transition from the outcomes of evidence-based research and research synthesis, documented in well-crafted systematic reviews, to evidence-based clinical decision-making for patient-centered, effectiveness-focused and evidence-based treatment intervention and patient follow-up.

Methotrexate (MTX) is often the drug of choice to help control psoriasis, and PsA. It is generally considered as a disease-modifying anti-rheumatic drug (DMARD), MTX administration - generally 7.5 mg to 25 mg once a week - alleviates symptoms, attenuates disease activity, and prevents progression of psoriasis, and PsA by slowing down the replication of skin cells and suppressing inflammation. MTX, formerly known as amethopterin, is a pterin derivative that mimics the folic acid coenzyme, and blocks DNA and RNA synthesis by inhibiting tetrahydrofolate synthesis [50], thus blunting cycle progression. MTX has relatively common, though principally mild side-effects, principally in terms of gastrointestinal toxicity [51].

In the context of testing the effectiveness and possible harmful effects of MTX in alleviating symptoms, attenuating disease activity, and preventing progression of PsA, a recent systematic review with meta-analysis studied a bibliome of 8 randomized controlled trials and quasi-randomized controlled trials across Europe, North America and Asia. The patient sample age varied from 26 to 52 years of age; duration of PsA varied from 1 to 9 years; and MTX treatment dose varied from 7.5 mg to 25 mg orally per week for up to six months. Disease severity, assessed by the continuous measure of PsA response criteria, demonstrated an absolute difference of 16% more responders in the MTX, compared to the Placebo group, with a number-needed-to-treat for an additional beneficial outcome of 6 (CI95=5-25), with an overall risk ration of MTX side-effects of 1.32 (CI95= 0.51-3.42). Benefits of MTX treatment on PsA radiographic disease progression, enthesitis or dactylitis, and risks of MTX intervention in terms of fatigue, gastrointestinal disturbances, and other less frequent side-effects beyond a 6-month course of treatment, at higher-dose of MTX, or with joint treatment protocols (e.g., MTX and cyclosporine) were not examined in the study [52].

In contrast, the efficacy of MTX in treating PsA could not be demonstrated in a separate systematic review consisting of a bibliome of 7 randomized controlled trials, except when MTX intervention was supplemented with immune suppressants (e.g., TNFα-inhibitor [e.g., infliximab, adalimumab or certolizumab], or the calcineurin inhibitor, cyclosporin A). In all cases, combination therapy was superior to MTX monotherapy, although MTX alone appeared to have some benefits for psoriasis without arthritic manifestations [53]. Moreover, another recent systematic review of 39 randomized controlled trials of oral, subcutaneous or intramuscular MTX, at a start dose of 7.5 mg/week to 15 mg/week, reported a general lack of high-quality evidence in support of MTX for treating adult patients with psoriasis, in large part because of the dearth of homogeneity with respect of MTX [54].

In brief, the complex nature and etiology of psoriasis and PsA, the varied treatment protocols and MTX dosages, lead to inherent methodological difficulties in obtaining the best available definitive evidence in systematic reviews with, or without meta-analysis. In general, the reported studies are "living" systematic reviews, meaning that the research synthesis is updated regularly as new clinical studies are published. The net result of that process, which favors the regular update of the beat available evidence, is that some updates may strengthen or weaken previously obtained conclusion.

The current (2022/3) best available evidence indicates that immune suppressing monoclonal antibodies directed against IL17, IL12/23, IL23 alone or TNFα, either alone or in combination with non-biological systemic DMARD's (e.g., MTX or Cyclosporin-A) are more efficient than DMARD's alone in treating patients with moderate-to-severe psoriasis and PsA, although these immune suppressive interventions may have heightened risk of side-effects. The general consensus, therefore, is that, at this stage of research synthesis, the outcomes of systematic reviews with, or without meta-analysis, be they final or living [47, 48], have to be viewed with caution, before definitive evidence can be established on the long-term benefits and safety of MTX, alone or in combination with specific immune modifiers (i.e., targeted monoclonal antibodies) for patients with psoriasis and PsA [55].

In conclusion, psoriasis and its sequelae, including PsA, are complex autoimmune entities with general chronic inflammation of multi-factorial etiology, including stress in its myriad manifestations [56]. Evidence-based modalities, the seamless transition from the outcomes of evidence-based research and research synthesis, documented in well-crafted systematic reviews, to evidence-based clinical decision-making for patient-centered, effectiveness-focused and evidence-based treatment intervention and patient follow-up, are more evident in the treatment of psoriasis than of PsA at this point in time. Continued concerted research toward the elucidation of the fundamental immunopathology of psoriasis and PsA is as timely as it is critical.
